# Modelling the regenerative niche: a major challenge in biomaterials research^†^

**DOI:** 10.1093/rb/rbv018

**Published:** 2015-10-14

**Authors:** C. James Kirkpatrick

**Affiliations:** ^1^REPAIR-Lab, Institute of Pathology, University Medical Center, Johannes Gutenberg University Mainz, D-55101 Mainz, Germany;; ^2^Department of Biomaterials, Sahlgrenska Academy, University of Gothenburg, Gothenburg, Sweden

**Keywords:** biomaterial–cell interaction, regenerative mechanism, stem cells

## Abstract

By definition, biomaterials are developed for clinical application. In the field of regenerative medicine their principal function is to play a significant, and, if possible, an instructive role in tissue healing. In the last analysis the latter involves targeting the ‘regenerative niche’. The present paper will address the problem of simulating this niche in the laboratory and adopts a life science approach involving the harnessing of heterotypic cellular communication to achieve this, that is, the ability of cells of different types to mutually influence cellular functions. Thus, co-culture systems using human cells are the methodological focus and will concern four exemplary fields of regeneration, namely, bone, soft tissue, lower respiratory tract and airway regeneration. The working hypothesis underlying this approach is that *in vitro* models of higher complexity will be more clinically relevant than simple monolayer cultures of transformed cell lines in testing innovative strategies with biomaterials for regeneration.

## The concept of the ‘regenerative niche’

In regenerative medicine (RegMed) the underlying principle involves taking interventional measures to stimulate innate healing mechanisms, which intimately involves the immune system [1]. This presumes a firm understanding of the latter. However, even a cursory glance at human pathology indicates that there are organs, such as the heart and central nervous system (CNS), which in the adult organism have very limited or no clinically significant regenerative capacity, despite possessing like all other organs stem or progenitor cells [2]. Furthermore, our knowledge of the biological background to this failure to repair is minimal. In the last analysis we can reduce repair processes to the function of a supposed ‘regenerative niche’, present in every tissue type and responsible for an attempted ‘*restitutio ad integrum,*’ or a return to an anatomical and physiological normal state, that is, maintaining homeostasis. In repair processes it is becoming apparent that the outcome is markedly influenced by the interaction of immune cells, local stromal cells and matrix in the microenvironment of the damaged tissue [3, 4].

**Figure 1.**
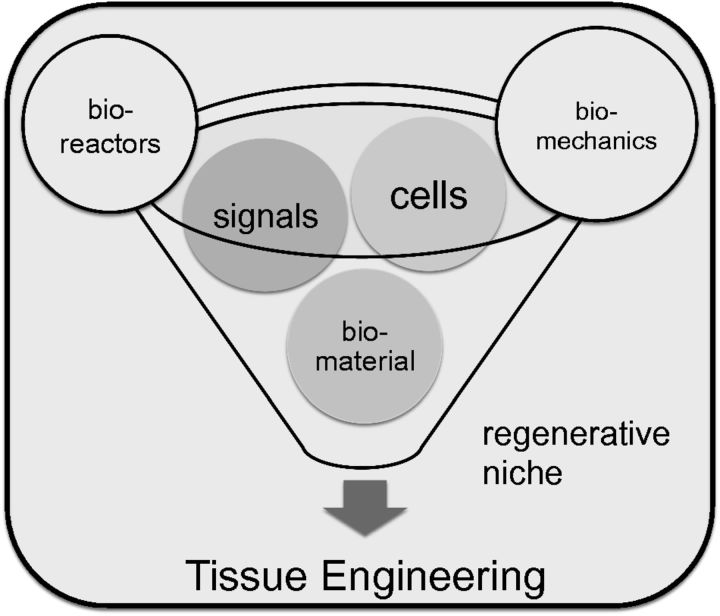
The classical tissue engineering paradigm with the three major components of biomaterial, bioactive signal molecules and relevant cells. Biomechanical considerations and the practicality of using bioreactors for seeding technologies represent corner-stones of this approach to regeneration.

The well-known tissue engineeering (TE) paradigm, consisting in its maximal form of a biomaterial, biological signals and cells, takes on added complexity in the context of bioreactors, which are of major importance for pre-seeding technologies and pre-conditioning of a TE construct [5–8]. Related to bioreactors are the overall biomechanical considerations operative in all living systems, as every cell of the body is subject to some form of biomechanical stress. Thus, it is with this background in mind that the regenerative niche needs to be viewed (Fig. 1).

**Figure 2.**
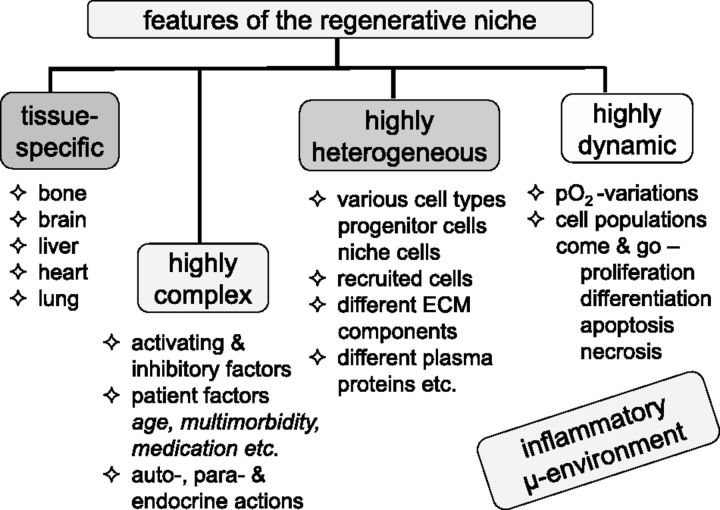
Features of the ‘regenerative niche’. Four sub-headings are used to characterize the regenerative niche, namely, its tissue-specificity, its high degree of complexity, its heterogeneity and its dynamics. A major influence on the regenerative niche is exerted by mediators released during the inflammatory response.

It is instructive to summarize the essential features of the regenerative niche (Fig. 2). First, it is tissue-specific, so that, whilst general principles concerning the functioning of a niche exist, the niche, for example, in the CNS is markedly different from that in the liver and so on. Thus, regenerative strategies for one may not necessarily apply to another. Second, it is highly complex, as its regulation is dependent on both activating and inhibitory factors, which may have autocrine (acting on the same cell), paracrine (acting on a neighbouring cell of different phenotype) or endocrine effects (acting via the circulation on a distant tissue) [9]. Added to this is the influence of patient age, the presence of a variety of accompanying diseases (multimorbidity) and the possible interference with the regenerative process by medication. Third, the regenerative niche is highly heterogeneous. Thus, in addition to local progenitor cells and the associated niche cells there are site-specific differences in extracellular matrix (ECM) components. It is evident that this variety of macromolecules and cellular types will be enhanced following tissue injury, as inflammatory cells and numerous activated components of the plasma protein cascade systems, for example, complement cascade, the coagulation and fibrinolytic systems, will be recruited and will markedly complicate the task of unravelling molecular mechanisms. Finally, fourth, the regenerative niche is highly dynamic, with oxygen levels fluctuating according to the state of integrity of the vascular systems, and cell populations increasing and decreasing, as activating and inhibitory signal molecules are released. Population control is exerted by a number of processes, including proliferation, differentiation, apoptosis and even necrosis, the latter being especially favoured by inadequate vascularization and infection.

## Modeling the ‘regenerative niche’: bone regeneration

It is evident from what has been discussed above that the complexity of the regenerative niche places the experimentalist in a difficult situation with respect to establishing reproducible models which, first, adequately represent essential features of the chosen niche, and, second, are not so complex that too many variables in the system make interpretation of the generated data impossible [10]. One solution to this problem in the *in vitro* arena is the use of heterotypic co-culture models [11]. It should be noted at this stage that the modelling of the regenerative niche in experimental models *in vivo*, although absolutely essential to our understanding, will not be a topic of the present paper.

Heterotypic co-culture models involve the culturing of more than one cell phenotype in a system which permits communication between the different cell types, so called ‘cellular crosstalk’ [12]. Most available co-culture models involve two cell types, but an example of a triple co-culture, featuring three different phenotypes, will be given. For TE applications this cellular crosstalk must also be investigated in the context of biomaterials, including a 3D configuration [13].

One of the fields of TE endeavour in which co-culture methodology has proved useful is the study of vascularization, which remains one of the major restrictions in regenerating large defects, as an adequate blood vessel network is essential to provide oxygen as well as nutrients and also to remove metabolic products from the tissues. We have carried out numerous such studies on mechanisms of cellular crosstalk in bone vascularization in the presence or absence of biomaterials, mostly in the form of 3D microporous scaffolds, such as silk fibroin [14, 15] and SPCL, a blend of starch and poly(caprolactone) [16, 17]. The two basic cell types for such co-cultures are human osteoblasts (OB) and human endothelial cells (EC), the latter being of two possible types, namely from the microvasculature (most often skin) [18] and the stable endothelial phenotype (OEC = outgrowth EC) from endothelial progenitor cells (EPC) formed in the bone marrow and released into the circulation [19, 20].

**Figure 3.**
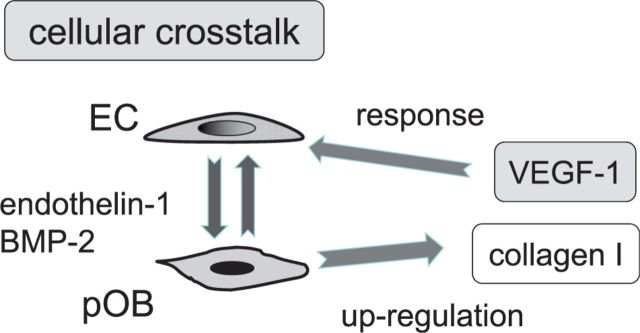
The phenomenon of ‘cellular crosstalk’ in the context of bone vascularization. Two major cellular players are the endothelial cell (EC) and the osteoblast (OB). In co-cultures of primary human OB (pOB) and microvascular EC osteoblasts can be stimulated by, for example, endothelial-derived endothelin-1 and BMP-2, which cause osteoblastic up-regulation of matrix signals, such as collagen I and growth factor signals, such as VEGF-1. The EC in turn react to these signals by switching to the angiogenic phenotype.

Fig. 3 summarizes essential features of the nature of this cellular communication, which in simple terms involves a mutual stimulation of the cell types resulting in biological signal production which facilitates neoangiogenesis or the formation of new microvascular blood vessels. Endothelin is synthesized in EC [21]. Basically the EC stimulate the OB via molecules like endothelin-1 and BMP-2 (bone morphogenetic protein-2) to upregulate collagen type I [22, 23], an essential matrix macromolecule, and VEGF (vascular endothelial growth factor) [24], one of the most important pro-angiogenic growth factors, to which the EC then respond by proliferation, migration and formation of tubes or sprouts (CLS = capillary-like structures). The most surprising feature of this co-culture was that the CLS formation reached a high plateau phase between weeks 3 and 5 and still showed clear viability in the complete absence of any exogeneously added pro-angiogenic GFs [25]. Under similar culture conditions EC alone would not survive beyond a 10d period.

Subsequent experiments demonstrated that this cellular crosstalk was also possible between the progenitor cells of both cell phenotypes [26]. Thus, human bone marrow mesenchymal stem cells (MSC), driven by the addition of β-glycerophosphate, ascorbic acid and dexamethasone to the osteogenic lineage, co-cultured with OEC from human peripheral blood EPC also yielded similar lumen-containing CTS, provided that the medium for the co-culture was changed from osteogenic differentiating medium to an endothelial growth medium. This result from two progenitor cell types, which in clinical practice could derive from a single patient, is significant for a pre-seeding step in a TE construct.

During tissue injury blood vessels are often damaged so that a state of low oxygen partial pressure (hypoxia) can ensue. Changing oxygen levels are therefore highly relevant for regenerative processes and require consideration in our theoretical and experimental considerations. In co-cultures of human OEC and OB we discovered that continuous hypoxia (2% oxygen) failed to stimulate vascular sprout formation. By contrast, repeated short-term (up to 8 h duration) did elicit an upregulation of sprout formation in the co-culture (Li et al. 2015; manuscript submitted)

A histopathological view of injured tissue, regardless of whether it is hard or soft tissue, also underlines the presence of inflammatory cells, which in the first days is predominantly granulocytic, but then changes to being monocyte/macrophage-dominated. This raises the question concerning their role in the mechanisms of the healing process and prompted us to attempt a triple culture, fully conscious of the fact that adding a third cell type would make the experimental constellation much more complex. To simulate the early phase of bone regeneration the focus was placed on the pro-inflammatory phenotype of the macrophage, a model of which can be experimentally established with the THP-1 monocytic cell line, treated with a low dose (8 nM for 4 days) of phorbol myristate acetate (PMA). The double co-culture, consisting of human OB and OEC, was compared with the triple co-culture, which was contained the M1 macrophage phenotype [27]. At both gene transcription (qRT-PCR) and protein (ELISA) level, VEGF was statistically significantly upregulated in the triple compared to the double co-culture after 14 days. Confocal laser scanning microscopical studies demonstrated a marked difference in CLS formation even after 7 days and was confirmed by computer-assisted image analysis to be statistically significantly higher in the triple co-culture set-up.

## Modeling the ‘regenerative niche’: soft tissue regeneration

This behaviour of relevant cells for vascularization in the regenerative niche of bone led subsequently to a series of experiments to test the hypothesis that a similar cell cooperativity might be operative in soft tissue. Thus, in the co-culture model osteoblasts were replaced by fibroblasts and the human skin taken as tissue origin for both cell types. Thus, human dermal microvascular EC (HDMEC) were co-cultivated with human dermal fibroblasts (HDF). As biomaterial a compressed collagen was chosen, as this was only 12% hydrated, as *in vivo*, compared with the unphysiological >95% hydration of conventional collagen gels for most *in vitro* models [28]. Seeding HDMEC alone on top of the compressed collagen gel led to the formation of an intact EC monolayer, without any penetration of the gel and without any evidence of an angiogenic phenotype. However, seeding the EC on top with simultaneous incorporation of HDF into the gel resulted in the formation of lumen-containing vascular structures within the gel just below the level of the endothelial monolayer on top [29]. Immunohistochemical studies confirmed the presence of collagen type IV and laminin as essential components of the basement membrane on the abluminal aspect of the CLS. One major advantage of the use of compressed collagen as a biomaterial for TE is the fact that, despite marked colonization of the gel by fibroblasts, gel contraction did not occur. This would suggest that contractures could be avoided—one of the severe aesthetic sequelae of contracture formation in the skin. However, this still has to be confirmed in relevant *in vivo* studies.

## Modeling the ‘regenerative niche’: lower respiratory tract regeneration

The lung is one of the most complex organ systems in the body. The corollary of this is that establishing relevant models of regenerative niches in the human lung is far from trivial. The characteristic entity of the lower respiratory tract is the alveolus with the air–blood barrier (ABB), the epithelial–endothelial interface across which gaseous exchange takes place and whose physiological function is essential for maintenance of blood oxygenation levels. Intensive experimentation was necessary to isolate successfully the essential epithelial cells from the human alveolus (alveolocyte type II, AT-II) and the human pulmonary microvascualr endothelial cells (HPMEC) and grow them together in co-culture [30]. However, as AT-II cells can only be cultivated in primary culture it was evident that a suitable alternative is required to permit establishment of control and test groups for reproducible studies of the ABB. This has been achieved and subsequently validated using two alternatives. First, the permanent cell line of alveolocyte phenotype, NCI H441, can be co-cultivated on one side of a microporous (0.4 µm) polycarbonate membrane with HPMEC on the opposite aspect of the membrane [31]. Second, H441 can be co-cultured with ISO-HAS-1 as microvascular endothelial phenotype [32], yielding a model of the ABB with which nanoparticle uptake and transport can be tested [33, 34]. Recently, we have been able to study the effect of surfactants on such interactions, this being very relevant for the *in vivo* situation, as under physiological conditions surfactant is present in the alveoli [35]. An additional level of complexity arises from the necessity to investigate the role of the macrophage, as this important phagocyte is also resident in the alveoli. This has been achieved in a triple culture, in which the ABB double co-culture is augmented on the epithelial side by macrophages of different polarization [36].

A major challenge for the future is how to develop translatable regenerative strategies for the lower respiratory tract. This basically involves targeting the AT-II, as this is the cell with the regenerative capacity for the epithelial side and/or HPMEC, as the latter is the partner cell on the pulmonary circulation side of the ABB.

## Modeling the ‘regenerative niche’: airway regeneration

The upper respiratory tract and especially the large airways (trachea, bronchi) are not only of academic interest for RegMed but also of great clinical significance, as damage to these structures can be a consequence of severe inflammatory conditions such as Wegener’s granulomatosis or polychondritis, making it necessary to find a replacement or regenerative strategy [37, 38].

There are two principal areas of investigation whose input is essential for successful clinical translation in any strategy for Tissue Engineering. In the first place, the human progenitor cells should be reliably isolated and cutivated under those circumstances needed to yield a functional respiratory mucosa as well as mural components (smooth muscle cells (SMC) and cartilage. On the other hand, such cell biological knowledge and existing tool-box are futile if the biomaterial with optimal physical and chemical characteristics to reconstruct the airways is unavailable. Concerning the latter, a very promising scaffold is the decellularized form of an airway, for example, of allogenic or xenogenic origin [39].

**Figure 4.**
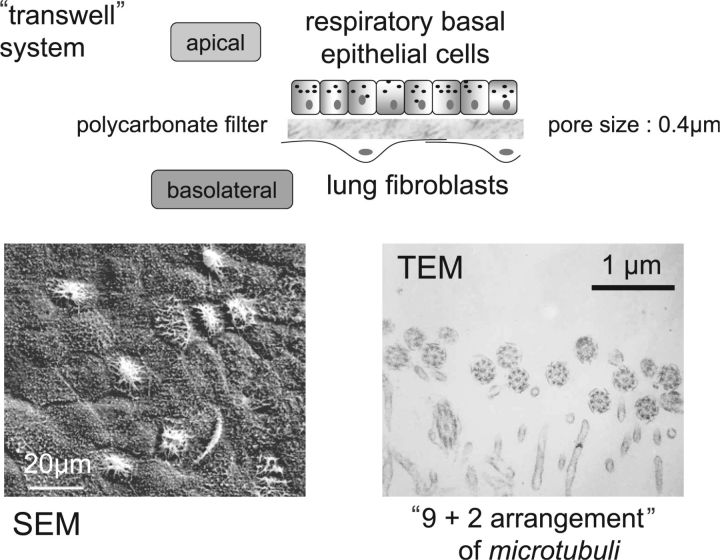
Cellular crosstalk in the context of airway regeneration. Using the commercially available Transwell® system, consisting of a synthetic polymer insert with a polycarbonate membrane for use in 24 multiwell tissue culture plates, human respiratory basal epithelial cells can be co-cultured with human lung fibroblasts (either primary adult or the foetal cell line Wi-38). The culture must be conducted under so-called ‘air-lift’ conditions, in which the epithelial layer is in contact with a thin film of medium, but well exposed to air, otherwise differentiation to a complete respiratory mucosa will not take place. The scanning electron micrograph (SEM) illustrates the well-formed monolayer of epithelial cells containing a sub-population of ciliated cells. Transmission electron microscopy (TEM) confirms the genuine physiological structure of the cilia with the so-called ‘9 + 2’ arrangement of the microtubuli in the cilia.

With respect to the generation of the cellular components it has become apparent that there are site-specific regenerative niches in the course of the pulmonary tree [40]. Together with suitable biomaterial scaffolds this forms the basis for translatable TE [41]. In our own research we have succeeded in generating a functional respiratory mucosa by enabling cellular cooperativity in a co-culture of lung fibroblasts and basal epithelial cells from the human bronchial system (Fig. 4), as the latter represent the corresponding progenitor cell phenotype [42]. The resulting mucosa contains a subpopulation of progenitor cells, mucus-producing cells and ciliated columnar epithelial cells with functioning cilia (Fig. 4), which beat with the same frequency as *in vivo*. However, the epithelial layer is only the innermost portion of the airway. The mural structures also require adequate regeneration, thus involving, amongst others, fibroblasts, SMC, EC and chondrocytes. Using decellularized porcine airway as a scaffold initial studies with relevant cell lines indicate that epithelial cells, fibroblasts and EC in a triple culture system can indeed colonize the natural scaffold [43]. However, much more intensive research is required to develop the regenerative strategy which will be feasible in a translational setting. A theoretical possibility would be to use a co-culture of autologous basal epithelial cells and MSC on a decellularized scaffold. In addition to the cellular challenges there is also the requirement for mechanical stability of the airway unit and integration of any regenerated segment into the pulmonary tree. Moreover, although the MSC could give the chondrogenic phenotype, there is the existing problem of its stability in the long-term, as the ‘default’ phenotype seems to be the osteogenic lineage, that is, cartilage formed from MSC can often become hypertrophic and facilitatae calcium deposition [44].

There are numerous other regenerative niches which can be modelled in suitable cell co-culture systems. However, space does not permit a detailed discussion. Finally, a brief look at some of the clinically relevant challenges for the future are worthy of attention. The models which have been established *in vitro* are generally physiological models in the sense that they represent a system which simulates some structural and/or functional aspects of the niche *in vivo*. To be regarded as a valid scientific model it is generally required to show that what is established in the laboratory has major characteristics of what is observed in the physiological situation *in situ*. However, the fact is usually ignored that a regenerative strategy is applied not to a physiological, but rather a pathological scenario. Hence, the model systems adopted should also reflect the latter. The ‘real life’ situation has been partially addressed at the end of the section on bone regeneration (*vide supra*) with reference to a frequent hypoxic state as well as the inflammatory microenvironment, which we have attempted to simulate in a triple culture model incorporating the pro-inflammatory phenotype of the macrophage [27].

However, the initial inflammatory reaction is a physiological response intended to initiate healing processes. However, if this is protracted, and especially if infection is super-imposed, regeneration then has to proceed in a pathological microenvironment. In fact, RegMed applications in general are conceived for a broad spectrum of clinical situations which could be termed ‘hostile environments’. These include post-traumatic scenarios, in which there is widespread tissue damage, leading to *tissue defects* of varying proportions, post-operative situations in cancer patients where there is a high risk of *residual cancer*, and the common prevailing condition in older patients, namely *multimorbidity*. In the latter case there are often healing defects, which in older patients can be partially attributed to poorly functioning regenerative niches, in addition to poor tissue perfusion and a likelihood of infection. These can be common problems if one of the underlying conditions is diabetes mellitus [45]. Added to all of these complicating scenarios are the widely unknown effects of medication, as patients may be on long-term adminstration of more than one drug. Even a cursory glance at these additional factors leads to an awareness that the scientific community has just begun to tackle some of the challenges in TE and RegMed. Special emphasis needs to be placed on unravelling the mechanisms of regeneration within the scope of relevant pathological processes.
